# Transcriptomics of differential vector competence: West Nile virus infection in two populations of *Culex pipiens quinquefasciatus* linked to ovary development

**DOI:** 10.1186/1471-2164-15-513

**Published:** 2014-06-22

**Authors:** Dongyoung Shin, Ayse Civana, Carolina Acevedo, Chelsea T Smartt

**Affiliations:** Florida Medical Entomology Laboratory, University of Florida, 200 9th St. S.E., Vero Beach, FL 32962 USA

**Keywords:** *Culex pipiens quinquefasciatus*, West Nile virus (WNV), High throughput RNA sequencing, Vector competence

## Abstract

**Background:**

Understanding mechanisms that contribute to viral dissemination in mosquito vectors will contribute to our ability to interfere with the transmission of viral pathogens that impact public health. The expression of genes in two *Culex pipiens quinquefasciatus* populations from Florida with known differences in vector competence to West Nile virus (WNV) were compared using high throughput sequencing.

**Results:**

A total of 15,176 transcripts were combined for comparison of expression differences between the two populations and 118 transcripts were differentially expressed (p < 0.05). The fold change in expression of the differentially expressed genes ranged from -7.5 – 6.13. The more competent population for WNV (Gainesville) over expressed 77 genes and down regulated 44 genes, compared with the less competent population for WNV (Vero Beach). Also, splicing analysis identified 3 transcripts with significantly different splice forms between the two populations. The functional analysis showed that the largest proportion of transcripts was included in the catalytic activity and transporter activity groups except for those in the unknown group. Interestingly, the up- regulated gene set contained most of the catalytic activity function and the down- regulated gene set had a notable proportion of transcripts with transporter activity function. Immune response category was shown in only the down regulated gene set, although those represent a relatively small portion of the function. Several different vitellogenin genes were expressed differentially. Based on the RNAseq data analysis, ovary development was compared across the populations and following WNV infection. There were significant differences among the compared groups.

**Conclusions:**

This study suggests that ovary development is correlated to vector competence in two *Culex* populations in Florida. Both populations control energy allocations to reproduction as a response to WNV. This result provides novel insight into the defense mechanism used by *Culex* spp. mosquitoes against WNV.

## Background

West Nile virus was successfully introduced to the Western Hemisphere in 1999 and has since spread westward across the United States, southward into Central America and the Caribbean and northward into Canada [1]. West Nile virus (WNV) is transmitted to humans by the bite of mosquitoes that previously acquired the virus by feeding on infected birds. Since WNV was detected in New York in 1999, the CDC has reported over 39,000 human cases [2, 3] and has been detected in 59 mosquito species throughout North America, although less than 10% are considered principal WNV vectors [4]. Mosquitoes of the genus Culex are the primary vectors of WNV in the US due to their vector competence and host preference [5]. There is geographic variation in Culex WNV vectors, due to the presence of different, locally important *Culex* spp.; such as *Culex pipiens pipiens* in the Northeast, *Cx. tarsalis* in the West, and *Cx. p. quinquefasciatus* and *Cx. nigripalpus* in the South. Additionally, refractoriness can be attributed to the type of virus and variation in the genetics of the virus, which can affect an individual mosquito or mosquito species. Different populations of the same mosquito species may also show differential vector competence under similar environmental conditions [6–9] due to genetic differences contributing to vector competence [10]. Several studies have shown that local populations of *Culex* spp. vary in their capacity to transmit WNV [11, 12]. Recent studies have supported and extended the concept by investigating certain aspects of vector competence including susceptibility of different mosquito species to WNV, permissiveness of various mosquito tissues to infection, and release of virus from infected salivary glands into the saliva [13, 14]. Differences in vector competence could be attributed to barriers to infection, including midgut infection, midgut escape and salivary gland invasion barriers [15, 16], as well as the effects of extrinsic and intrinsic factors [17–19]. Vector competence is also likely affected by the innate immune response, which can result in gene expression differences [20–22].

Although the work cited above proposes that vector competence involves complex interactions between arbovirus and mosquito, the differences in the ability of one population to disseminate WNV better than another population was not addressed nor was the modification in competence attributed to changes in the transcriptome. For example the work by Sanders et al. [23] reported alphavirus-induced mosquito midgut transcription changes in genes involved in vesicle transport and terminal kinase immune cascades, but stopped short at assigning them a role in alphavirus infection and/or dissemination. Mercado-Curiel et al. [24] discovered two midgut cell proteins that bind all four serotypes of dengue virus. These proteins have been linked to *Aedes aegypti* vector competence for dengue virus [25]. Although these findings represent a step in understanding vector competence, the expression of these molecules post-virus exposure was not shown, and their potential as antiviral molecules was only shown in tissue culture.

The study demonstrates the relationship between the differing vector competence of two populations of *Culex p. quinquefasciatus* and gene expression changes that result from exposure to WNV. We know that these populations differ in their dissemination rates for WNV even when infection rates are the same [19] therefore changes in gene expression can be attributed to WNV infection processes after initial infection. Findings such as these would support our hypothesis that the difference in dissemination rates may be due to one of the infection barriers, such as midgut escape, in the less competent population. However this study extends this theory to include differences in the mosquito body transcriptome that might influence antiviral immune response, transcription and translation machinery sequestration and which may ultimately lead to modification of dissemination and proposes the involvement of other organ-tissues in their vector competence differences. These findings are expected to help elucidate a potential arbovirus vector competence mechanism.

## Results and discussion

### Sample preparation and RNA sequencing

The expression of genes from two *Culex pipiens quinquefasciatus* populations [Vero Beach (CQV) and Gainesville (CQG)] from Florida with known differences in vector competence to West Nile virus (WNV) were compared using high throughput sequencing. The previous vector competence study with the same WNV infected CQV and CQG populations indicated significant difference with 44% and 82% dissemination rate, respectively, after 12 days of exposure [19]. In this study, four-day-old female mosquitoes were fed a blood meal containing 5.3 log10 pfu/ml of WNV. The titration of WNV in 6 mosquitoes in each population was tested (Table 1). The titer of the freshly fed mosquitoes was significantly lower than in the blood meal, but was not significantly different between mosquito populations indicating that genes that are differentially expressed between the two mosquito populations will not be attributable to differences in the amount of virus imbibed (Table 2). By 5 dpi, titer in the two populations increased to the same level as the blood meal, suggesting WNV replication. Five days following infection, a time that falls within the extrinsic incubation period for WNV of 3-6 days [26], RNA from female mosquito bodies was extracted and sent for transcriptome analysis using Illumina high throughput sequencing by the Sequencing Core in the Hussman Institute for Human Genomics, at the University of Miami. A total six RNA-seq libraries including triplication of each population were generated and sequenced (Table 2). A total 220,388,641 and 202,416,138 reads were generated from Vero Beach and Gainesville populations and a total of 88,402 transcripts hit on annotated transcript sequences of *Cx. quinquefasciatus* (Table 2). The data from each replicate was combined for differential analyses.

**Table 1:** **WNV titer in pooled mosquitoes from two populations (Vero Beach vs Gainesville) of** ***Cx. quinquefasciatus***

Population	Initial dose in bloodmeal (log PFU/ml)	Freshly fed (log PFU/ml)	5 days post infection (log PFU/ml)
CQV (Vero Beach)	5.3	3.7	5.4
CQG (Gainesville)	5.3	3.9	5.2

Titers were determined in mosquitoes (n = 6) that were either freshly fed or collected 5 days post-infection. WNV titer was also determined in the blood that was provided to the mosquitoes.

**Table 2:** **Mapping summary**

Population	Replicate	Total reads ^1^	No. transcripts ^2^
Vero beach	V1	70545050	14559
	V2	71274514	14119
	V3	78569077	14887
Gainesville	G1	72080828	15328
	G2	47154902	15139
	G3	83180408	14370

^1^Total number of sequences reads.

^2^Number of the annotated transcripts in *Culex quinquefasciatus* (Vectorbase) assembled with the sequence reads.

### Differential expression between WNV-infected *Cx. quinquefasciatus*females from two Florida populations

**Figure 1:**
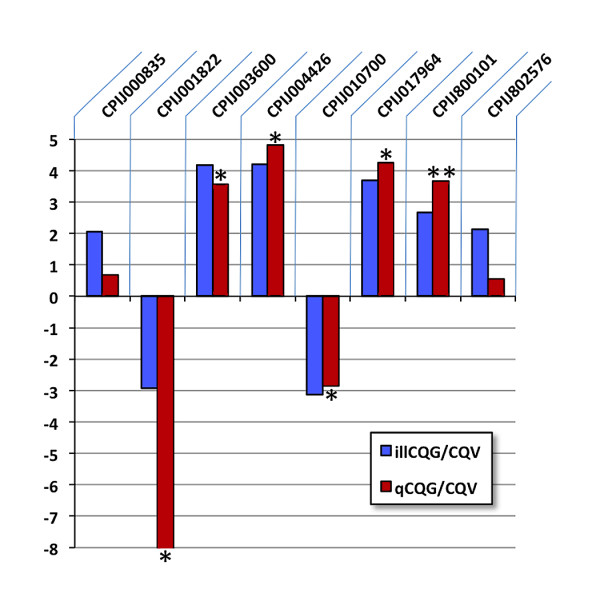
**Validation of the expression of transcripts between two** ***Cx. quinquefasciatus*** **populations by qRT-PCR.** *< 0.05. **< 0.01.

A total of 15,176 transcripts were combined for comparison of expression differences between the two populations and 118 transcripts were differentially expressed (p < 0.001) (Table 3). Of these, 41 transcripts showed decreased expression in CQG compared to CQV, and 77 transcripts increased abundance in CQG compared to CQV, 5 days after a WNV-infected blood meal. In order to validate the RNAseq data analysis, several genes were randomly selected and their gene expression level tested by quantitative real time PCR. All the selected genes showed significantly different expression levels between the two CQ populations, supporting the RNAseq data analysis (Figure 1).

**Table 3:** **List of differentially expressed genes between two populations related to WNV infection**

Transcript ID	Description	log2 (fold change)*
CPIJ019915-RA	alpha-glucosidase, putative	6.13
CPIJ019847-RA	ubiquinol-cytochrome c reductase complex 14 kDa protein	5.12
CPIJ005240-RA	latent nuclear antigen, putative	4.20
CPIJ004426-RA	conserved hypothetical protein	4.19
CPIJ003600-RA	ctl transporter, putative	4.18
CPIJ009603-RA	proto-oncogene tyrosine-protein kinase abl1	3.80
CPIJ017964-RA	trypsin 7 precursor	3.69
CPIJ020286-RA	isoleucyl tRNA synthetase, putative	3.43
CPIJ010313-RA	conserved hypothetical protein	3.24
CPIJ013626-RA	conserved hypothetical protein	3.07
CPIJ011888-RA	carbamoyl-phosphate synthase large chain	3.06
CPIJ014553-RA	salivary long D7 protein 3	2.78
CPIJ800101-RA	long form D7clu1 salivary protein	2.66
CPIJ015241-RA	alkaline phosphatase	2.64
CPIJ019718-RA	conserved hypothetical protein	2.62
CPIJ005238-RA	conserved hypothetical protein	2.55
CPIJ014551-RA	D7 protein precursor	2.44
CPIJ002236-RA	hypothetical protein	2.41
CPIJ801512-RA	cat eye syndrome critical region protein 1 precursor	2.41
CPIJ800099-RA	7.7 kDa salivary cysteine-rich peptide	2.37
CPIJ008464-RA	hypothetical protein	2.20
CPIJ012065-RA	tryptophan transporter	2.18
CPIJ018241-RA	microsomal glutathione S-transferase 1	2.13
CPIJ802576-RA	conserved hypothetical protein	2.13
CPIJ003205-RA	hypothetical protein	2.12
CPIJ005906-RA	conserved hypothetical protein	2.09
CPIJ008471-RA	hypothetical protein	2.09
CPIJ019221-RA	hypothetical protein	2.08
CPIJ015059-RA	conserved hypothetical protein	2.06
CPIJ000835-RA	chymotrypsin-2	2.04
CPIJ014970-RA	niemann-Pick C1 protein precursor	2.02
CPIJ800222-RA	cytochrome P450	2.02
CPIJ003807-RA	allantoinase	2.01
CPIJ002103-RA	conserved hypothetical protein	2.00
CPIJ018300-RA	salivary secreted antigen-5 precursor AG5-3	1.94
CPIJ018735-RA	D7 protein	1.93
CPIJ016362-RA	alpha-glucosidase precursor	1.93
CPIJ018565-RA	peptide methionine sulfoxide reductase	1.88
CPIJ014550-RA	long form D7Bclu1 salivary protein	1.86
CPIJ018736-RA	D7 protein, putative	1.86
CPIJ008977-RA	pyridoxal phosphate phosphatase	1.84
CPIJ007820-RA	actin binding	1.81
CPIJ017499-RA	hypothetical protein	1.79
CPIJ015057-RA	conserved hypothetical protein	1.77
CPIJ003982-RA	hypothetical protein	1.72
CPIJ002681-RA	glutathione S-transferase	1.72
CPIJ020056-RA	cysteine dioxygenase	1.69
CPIJ002737-RA	MPA2 allergen	1.69
CPIJ014970-RA	niemann-Pick C1 protein precursor	1.68
CPIJ002367-RA	conserved hypothetical protein	1.68
CPIJ003807-RA	allantoinase	1.68
CPIJ002668-RA	hypothetical protein	1.65
CPIJ001247-RA	conserved hypothetical protein	1.64
CPIJ007379-RA	hypothetical protein	1.63
CPIJ011998-RA	zinc carboxypeptidase A 1 precursor	1.63
CPIJ019944-RA	hypothetical protein	1.61
CPIJ010456-RA	cysteine dioxygenase	1.58
CPIJ800096-RA	41.9 kDa basic salivary protein	1.58
CPIJ016041-RA	hypothetical protein	1.54
CPIJ005910-RA	7.8 kDa basic salivary peptide, putative	1.54
CPIJ012398-RA	histone H3 type 2	1.54
CPIJ005741-RA	calmodulin-binding protein trpl	1.53
CPIJ010799-RA	carboxypeptidase B	1.50
CPIJ015715-RA	AMP dependent ligase, putative	1.47
CPIJ005463-RA	salivary lipase	1.47
CPIJ800229-RA	cytochrome P450 17A1	1.46
CPIJ016921-RA	3-oxoacyl-[acyl-carrier-protein] reductase 1	1.46
CPIJ006975-RA	vacuolar ATP synthase subunit g	1.46
CPIJ015700-RA	aquaporin	1.45
CPIJ011388-RA	diazepam binding inhibitor, putative	1.44
CPIJ016922-RA	serine 3-dehydrogenase	1.40
CPIJ003185-RA	gamma-glutamyltranspeptidase 1 precursor	1.39
CPIJ005064-RA	alpha-amylase precursor	1.37
CPIJ005786-RA	actin-2	1.37
CPIJ800225-RA	cytochrome P450	1.34
CPIJ015103-RA	trypsin-5 precursor	1.30
CPIJ010421-RA	60S ribosomal protein L24	1.24
CPIJ003274-RA	vacuolar proton translocating ATPase 116 kDa subunit a isoform 1	-1.30
CPIJ015839-RA	conserved hypothetical protein	-1.45
CPIJ802157-RA	nuclear hormone receptor FTZ-F1 beta	-1.61
CPIJ010767-RA	hypothetical protein	-1.64
CPIJ009700-RA	conserved hypothetical protein	-1.64
CPIJ800076-RA	small calcium-binding mitochondrial carrier, putative	-1.64
CPIJ015292-RA	zinc-finger protein DPF3	-1.68
CPIJ008221-RA	conserved hypothetical protein	-1.69
CPIJ800015-RA	Gustatory Receptor	-1.74
CPIJ015294-RA	dopamine receptor, invertebrate	-1.77
CPIJ800292-RA	cytochrome P450 2A12	-1.80
CPIJ010701-RA	cecropin	-1.85
CPIJ015936-RA	hypothetical protein	-2.05
CPIJ006159-RA	glutathione-requiring prostaglandin D synthase	-2.10
CPIJ016495-RA	thrombospondin-4, putative	-2.16
CPIJ010699-RA	cecropin A	-2.18
CPIJ016128-RA	conserved hypothetical protein	-2.23
CPIJ017192-RA	conserved hypothetical protein	-2.24
CPIJ005518-RA	conserved hypothetical protein	-2.26
CPIJ003470-RA	hypothetical protein	-2.37
CPIJ010191-RA	vitellogenin-A1 precursor	-2.45
CPIJ000056-RA	larval serum protein 1 beta chain precursor	-2.54
CPIJ010516-RA	phosphoenolpyruvate carboxykinase	-2.56
CPIJ005941-RA	ADP, ATP carrier protein 2	-2.57
CPIJ010190-RA	vitellogenin-A1 precursor	-2.64
CPIJ001820-RA	larval serum protein 2 precursor	-2.89
CPIJ009033-RA	arylphorin subunit C223 precursor	-2.89
CPIJ001822-RA	larval serum protein 2 precursor	-2.91
CPIJ003588-RA	ubiquitin	-3.00
CPIJ009506-RA	hexamerin 2 beta	-3.05
CPIJ007783-RA	arylphorin subunit alpha precursor	-3.05
CPIJ010552-RA	cyclin d	-3.11
CPIJ010700-RA	putative 4.2 kda basic salivary peptide	-3.12
CPIJ002311-RA	conserved hypothetical protein	-3.32
CPIJ013252-RA	conserved hypothetical protein	-3.57
CPIJ010553-RA	cyclin d	-3.68
CPIJ014871-RA	uridine cytidine kinase i	-4.26
CPIJ008969-RA	conserved hypothetical protein	-4.81
CPIJ013631-RA	conserved hypothetical protein	-5.35
CPIJ009149-RA	conserved hypothetical protein	-5.92
CPIJ008969-RA	conserved hypothetical protein	-7.51

*Fold change is the gene expression level change in Gainesville population after WNV infection compared to Vero Beach population log CQG/CQV.

Comparison of the expression of the118 transcripts with significant expression differences revealed that only 5 genes were not expressed in the CQV but had greater than 3 fold change in expression in the CQG population; while only 3 of the 118 genes were not expressed in CQG and were down regulated between -3 and -7 fold. Some of these genes were included in the validation by qRT-PCR (CPIJ003600 and CPIJ004426).

Investigation of transcripts with expression levels of less than 1 fold change in expression in either of the two *Cx. quinquefasciatus* populations was performed to determine if the differences in dissemination was due to absence of expressed products. Of the 5 transcripts expressed only in the more permissive population CQG, one transcript (CPIJ003600-RA) codes for a ctl transporter and its functional parental attribute suggest it is a cellular component involved in membrane transport and is 4-fold more highly expressed. Two transcription products, the first encoding a putative alpha-xylosidase (CPIJ019915-RA) and the second of unknown function (CPIJ010313-RA) have a role in catalysis and are 18-fold and 6-fold higher in expression in the CQG population, respectively. A transcript (CPIJ004426-RA) involved in binding or interacting with zinc is 4-fold higher in expression. Another transcript (CPIJ005240-RA) is novel and does not match any functional groups but is 17-fold higher in expression in the CQG population. The three transcripts expressed only in the less permissive mosquito population, CQV (CPIJ002311-RA, CPIJ013631-RA, CPIJ008969-RA), were novel and therefore functional parental group assignments could not be made.

### Functional analysis of differentially expressed transcripts by GO

**Figure 2:**
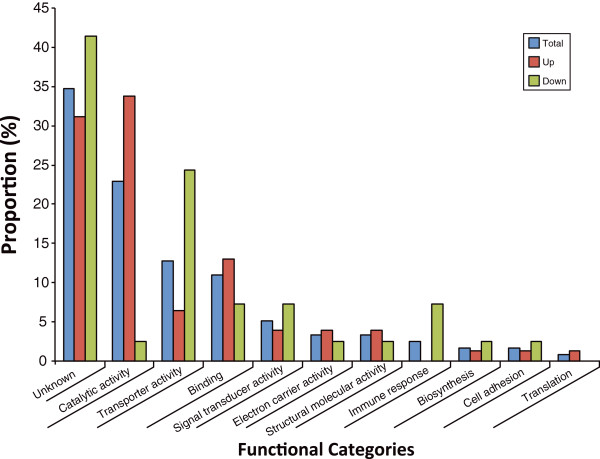
**Functional characterization of significantly differentially expressed transcripts between two** ***Cx. quinquefasciatus*** **populations.** *UP are up-regulated genes in CQG; Down are down-regulated.

Functional analysis using GO terms was conducted on all 118 transcripts with significant expression difference between the CQG and CQV *Cx. quinquefasciatus* populations. The largest proportion of the total number of differentially expressed transcripts (34%) was of unknown function (Figure 2). Of the transcripts that were up regulated in CQG, 31% were of unknown function; while 41% of the down-regulated transcripts were of unknown function. Of the 118 transcripts, 22% had a role in catalysis, where a large majority (33%) were up regulated in the more permissive mosquito population, CQG. Gene products with catalytic activity included those involved in blood digestion such as trypsin (CPIJ017964) and chymotrypsin (CPIJ000835) both showing >2 fold change in expression in the CQG population. There were a number of transcripts encoding proteins such as carboxypeptidase and kinase present and the expression of an alpha- glucosidase, an enzyme involved in sugar feeding (CPIJ019915), was 6-fold higher in the CQG population. There were a number of transcripts encoding salivary gland catalytic enzymes, such as salivary lipase and alpha amylase. A number of transcripts encoding glutathione S-transferase, with roles in detoxification of xenobiotics, were present with fold expression changes ranging from 1.7-2.0 in the more WNV-permissive population.

Most of the transcripts involved in signal transduction, transporter activity and immune response were down regulated in the CQG population, 7%, 24%, 7%, respectively, compared to the CQV population. Signal transduction pathways involve the binding of extracellular signaling molecules to receptors on the cell surface, which in turn activates intracellular molecules eliciting a physiological response. In mosquitoes, these pathways are integral parts of their physiology and include cascades that control the innate immune response, odorant detection, and gene activation. Of the 5% transcripts with signal transduction activity, 3% had increased and 7% had decreased expression in the CQG population. Of the transcripts with signaling function, three with up-regulated expression in the CQG population had fold change in expression ranging from 1.6 – 2.1, of these two were similar to hedgehog receptor activity proteins and one was similar to G-protein coupled receptor proteins. Three transcripts had approximately -1.7-fold change in expression and were involved in cell function or activity, such as regulation of transcription.

Proteins with transporter activity are involved in the movement of ions and small molecules into, out of or within a cell, or between cells. Of the transcripts differentially expressed between the two WNV-infected *Cx quinquefasciatus* populations assigned the transporter activity GO term, 24% were down-regulated in CQG and 6% were upregulated. Transcripts with a 2-4 fold decrease in expression included those involved in lipid transport such as a vitellogenin precursor (CPIJ010191) and oxygen transport such as arylphorin subunit precursor (CPIJ007783, CPIJ009033) and larval serum protein 2 precursor (CPIJ001822). A number of transcripts with transport activity increased in expression, such as for transport of calcium, vacuolar and transmembrane transport, also a transcript involved in mitochondrial electron transport (CPIJ019847) showed a 5-fold increase in expression in the CQG population.

The immune response genes with fold change ranging from -3.0 to -1.8, are surprisingly all with similarity to the Cecropin family of antimicrobial genes of the innate immune response pathway.

Transcripts with binding, electron carrier, structural molecule activity, biosynthesis and cell adhesion GO term assignments were also represented, with 11% of the total number of transcripts, and 12% of the up-regulated transcripts, involved in binding. The other transcripts made up only ≤ 4%. Proteins involved in binding molecules could be used in transport of virus into cells (receptors) or in other molecular processes from blood detection to virus replication. Of the 11% with binding capabilities, 12% were upregulated in the CQG population, while 7% were suppressed in this population.

There were 7 proteins (CPIJ005910, CPIJ010700, CPIJ014550, CPIJ018300, CPIJ018735, CPIJ800096, and CPIJ800099) identified as having similarity to the salivary gland D7 precursor proteins with 1-3 fold increase in expression in the CQG population and similarity to odorant binding proteins (CPIJ014553, CPIJ800101, and CPIJ018736). Salivary gland products are known to be involved in defense mechanisms in mosquito biology and the 7 different salivary gland genes and immune response genes were differentially expressed in this study. Two transcripts assigned to binding had the highest fold expression. One transcript (CPIJ004426) with a 4-fold expression change was similar to a family of zinc ion binding proteins and another transcript (CPIJ013626) showing a 3 fold increase in expression was similar to chitin binding proteins. The over represented functional terms were analyzed with hyper geometric analysis with bonferroni correction (p < 0.05, Table 4), supporting the proportional functional analysis in Figure 2. Most of the GO terms from the differentially expressed genes were over represented in the functional enrichment analysis. The immune response function was not over represented in the differentially expressed gene list compared to the genome-wide functions but the over represented functions of “Detection of chemical stimulus involved in sensory perception of smell” and “odorant binding” identified in the dataset were associated with the immune response.

**Table 4:** **Over-represented functions in the differentially expressed genes between two populations**

GO term ID	Bonferroni p value	Functional description
GO:0000786	0	nucleosome
GO:0003779	0	actin binding
GO:0004180	0	carboxypeptidase activity
GO:0004611	0	phosphoenolpyruvate carboxykinase activity
GO:0004613	0	phosphoenolpyruvate carboxykinase (GTP) activity
GO:0004871	0	signal transducer activity
GO:0005215	0	transporter activity
GO:0005344	0	oxygen transporter activity
GO:0005525	0	GTP binding
GO:0005634	0	nucleus
GO:0005856	0	cytoskeleton
GO:0006122	0	mitochondrial electron transport, ubiquinol to cytochrome c
GO:0006207	0	‘de novo’ pyrimidine base biosynthetic process
GO:0006418	0	tRNA aminoacylation for protein translation
GO:0006810	0	transport
GO:0007155	0	cell adhesion
GO:0008081	0	phosphoric diester hydrolase activity
GO:0008121	0	ubiquinol-cytochrome-c reductase activity
GO:0008236	0	serine-type peptidase activity
GO:0015293	0	symporter activity
GO:0015629	0	actin cytoskeleton
GO:0016020	0	membrane
GO:0016471	0	vacuolar proton-transporting V-type ATPase complex
GO:0016705	0	oxidoreductase activity, acting on paired donors, with incorporation or reduction of molecular oxygen
GO:0016791	0	phosphatase activity
GO:0016874	0	ligase activity
GO:0016888	0	endodeoxyribonuclease activity, producing 5′-phosphomonoesters
GO:0019239	0	deaminase activity
GO:0043169	0	cation binding
GO:0046439	0	L-cysteine metabolic process
GO:0050911	0	detection of chemical stimulus involved in sensory perception of smell
GO:0019538	1.87E-26	protein metabolic process
GO:0043401	1.38E-19	steroid hormone mediated signaling pathway
GO:0004181	9.68E-19	metallocarboxypeptidase activity
GO:0016798	1.05E-17	hydrolase activity, acting on glycosyl bonds
GO:0005694	5.90E-11	chromosome
GO:0005216	1.30E-10	ion channel activity
GO:0006811	4.37E-10	ion transport
GO:0005886	1.89E-08	plasma membrane
GO:0005549	5.39E-08	odorant binding
GO:0006468	7.46E-08	protein phosphorylation
GO:0055085	6.75E-07	transmembrane transport
GO:0005488	4.67E-05	binding
GO:0007165	6.23E-05	signal transduction
GO:0016740	0.00016076	transferase activity
GO:0004872	0.00019634	receptor activity
GO:0016491	0.000216583	oxidoreductase activity
GO:0046872	0.004430445	metal ion binding
GO:0003824	0.040931204	catalytic activity

Cufflinks and Cuffdiff analyses measured the abundance of the expressed genes in each group and identified differences in Transcript Start Site (TSS) and alternative splicing in the two CQ populations (Table 5). In both the CQV and CQG populations, a total of thirteen transcripts showed different TSS. The expression ratio in different TSS of each transcript was calculated in the two CQ populations from Florida and compared. The expression of these genes from different start sites, we suggest, may be influenced by infection with WNV, although the transcripts do not show differential expression, and have some role in vector competence. Out of the total TSS, 5 transcripts were not annotated and the other 6 annotated transcripts were mostly related to binding to nucleus structure.

**Table 5:** **The list of isoform differences between two populations determined by transcript start site and splicing**

Locus	Description	log2 (fold-change)	p-value
TSS			
CPIJ010552	Cyclin d	−3.11246	1.89E-06
CPIJ010553	Cyclin d	−3.67618	3.57E-08
CPIJ010767	Hypothetical protein	−1.63552	1.66E-06
CPIJ001247	Conserved hypothetical protein	1.63872	2.17E-05
CPIJ013252	Conserved hypothetical protein	−3.5691	3.93E-06
CPIJ014249	Adenosine deaminase	2.90642	3.83E-05
CPIJ014553	Salivary long D7 protein 3	2.7805	2.88E-05
CPIJ019718	Conserved hypothetical protein	2.61949	8.63E-07
CPIJ020286	Isoleucyl tRNA synthetase, putative	3.42754	3.73E-05
CPIJ002737	MPA2 allergen	1.68586	4.67E-06
CPIJ003615	Salivary protein	2.6373	9.62E-06
CPIJ005240	Latent nuclear antigen, putative	4.20007	2.17E-06
CPIJ008546	Predicted protein	−2.95674	8.30E-06
Splicing			
CPIJ017916	Pdz	0.233522	1.00E-05
CPIJ009271	Vitellogenin	0.832555	1.00E-05
CPIJ009662	Tripartite	0.832555	1.00E-05

TSS: transcript start site.

Three transcripts showed different isoforms in CQV and CQG but their expression levels were not significant. The transcripts encode vitellogenin (CPIJ00927), tripartite (CPIJ00966), and pdz (post synaptic density protein, also known as Drosophila discs large tumor suppressor and zona occludens 1 protein, CPIJ01791). This vitellogenin is different from the other two vitellogenin precursors already identified in this study as significantly differentially down regulated but they all have the same lipid transport function. The tripartitie and pdz proteins play a role in pathogen recognition, including viruses, and cell signaling assembly, respectively.

As mentioned, there were two vitellogenin-A1 precursor genes differentially down regulated in CQG, -2.45 and -2.64. Another vitellogenin transcript was spliced differently between the two *Culex* populations. Vitellogenin genes, including vitellogenin-A1 in mosquitoes are known as egg yolk precursor proteins, which are used in ovary development and are regulated by juvenile hormone. The mosquito is known to synthesize vitellogenin in the fat body after a blood meal [27]. The fat body is one of the tissues in the mosquito that is well known to play a role in defense mechanisms. Results from this study suggest that the two populations showing different vectorial capacities appear to have functional differences in the lipid transport pathway between the fat body and ovary, and this difference might involve defense against WNV.

### Ovary development between the two CQ populations

**Figure 3:**
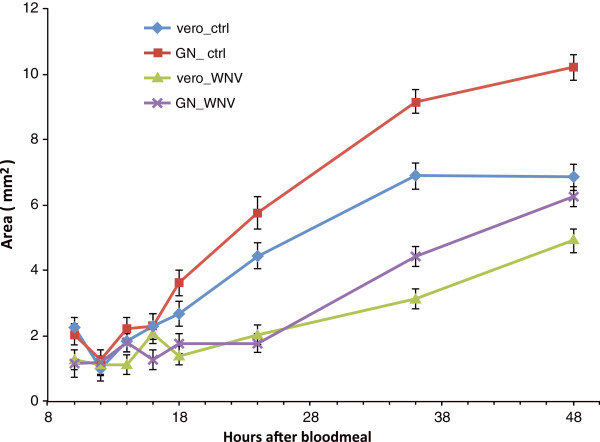
**Ovary growth after blood feeding.** Standard deviation is calculated from replicated samples tested at each time point and shows significantly different growth rates (ANOVA, *p <* 0.001). Vero = CQV; GN = CQG; ctrl = control.

We identified a number of vitellogenin precursors as differentially expressed genes between the two populations and also found different splicing forms between CGV and CQG. Generally vitellogenin is a precursor protein of lipoprotein and phospho protein in egg yolk and functions as a lipid transporter from fat body to ovary in invertebrates [27]. Ovary development allows increased reproductive capacity but results in a trade-off with allocation of resources in the fat body, impacting production of molecules that are essential for defense functions in insects [28]. Immune related genes and transport functions were enriched among differentially expressed genes in this study (Figure 2). Because of the correlation between the expression of vitellogenin and other co-regulated genes involved in defense and signal transduction as revealed in the RNAseq data, the size of the ovary in female mosquitoes from each population was measured after providing a blood meal, with or without WNV (Figure 3).

Female mosquitoes from both the CQG and CQV population significantly lessen the rate of ovary development around 18 hour post WNV infection. In the normal condition without WNV, CQG population forms larger ovaries than CQV but after the WNV infectious bloodmeal there is no significant difference between the two populations (Figure 3). This result agrees with the RNAseq data, where transport functions including vitellogenin are significantly decreased in CQG. This observation showed that *Culex* populations control the rate of ovary development when infected with WNV. Also, the WNV permissive population, CQG, appears to contribute more energy to reproduction in general. Once CQG is exposed to WNV, the CQG population alters the lipid transport to levels similar to the levels in CQV. Although there is no evidence about whether immune response genes influence ovary development or ovary development affects immune response, this decreased ovary development suggests that in CQ mosquitoes, vectorial capacity might result from the inability to properly route signals involved in lipid transport. The difference of ovary development rate before the WNV infection trial can be the influence of the vectorial capacity. Also, the drastic change in ovary development can be due to defense against WNV. These conclusions are speculative, at best, as no experiments have been done to directly measure resource allocation in these populations. Therefore there may be other underlying factors, such as a difference in dissemination rate, that contribute to the differences in ovary development in WNV infected mosquitoes in these populations.

## Conclusions

This study suggests that a correlation exists between ovary development and vector competence to WNV. Between two populations, the size of an ovary was significantly different 24 hours after bloodfeeding without the infection. This difference is almost impossible to notice when we observed these two populations during normal rearing under the same conditions. Interestingly, the size of the ovaries after WNV exposure was not statistically different between the two populations, supporting the RNAseq data that showed two vitellogenin precursor genes down regulating after WNV exposure. In mosquitoes, fat body and salivary glands play important roles in several immune pathways and in antimicrobial peptide production controlling infection by pathogens including virus. It has been demonstrated that a change in gene expression related to fat body and salivary gland function affects vectorial capacity [29, 30]. In the illumina data, a number of transcripts related to salivary proteins were also differentially expressed. This study suggests that energy allocation may affect defense mechanisms supported by transport function enrichment and warrants further study. The candidate genes including vitellogenin precursor, salivary protein, and immune response need to be tested by RNAi to verify the involvement in vector competence and to discover the pathway responsible for vector competence.

These results will help elucidate potential arbovirus vector competence mechanisms that will enhance our understanding of the genetic interactions between WNV and *Culex* spp. mosquitoes and help guide development of strategies for controlling the spread of vector-borne disease.

## Methods

### Mosquito sample preparation for RNAseq

Populations of *Culex pipiens quinquefasciatus* were collected from Gainesville, Florida and Vero Beach, Florida, then reared and maintained under standard insectary conditions (26°C, 14 h/10 h light/dark period, 70% relative humidity) and fed chicken blood for oviposition. The use of chickens to maintain colony mosquitoes described herein complies with the strict guidelines in the approved protocol (Project #201207682) awarded by the University of Florida Institutional Animal Care and Use Committee. Four-day-old female mosquitoes from two populations were fed on chicken blood (Hemostat, Dixon, CA) containing 5.3 log10 pfu/ml of West Nile virus. Five days following infection two hundred female mosquito bodies were collected and immediately frozen for RNA extraction. Extracted total RNA from each population was sent to the Center for Genome Technology Sequencing Core, John P. Hussman Institute for Human Genomics in University of Miami Miller School of Medicine for transcriptome analysis using Illumina high throughput sequencing. Six RNA-seq libraries were generated (Table 2). Cluster generation took place on the Illumina cBot according to the manufacturer’s recommendations. Sequencing took place on the Illumina HiSeq2000 using the reagents provided in the Illumina TruSeq PE Cluster Kit v3 and the TruSeq SBS Kit – HS (200 cycle) kit. Six RNAseq libraries were generated from the two populations of *Cx. quinquefasciatus,* showing different vector competence for WNV. Each of the replicates showed a consistent number of sequence reads and transcripts (Table 2). The reads were mapped on the Vectorbase *Cx. quinquefasciatus* database by using TopHat [31, 32].

The quantity of WNV RNA in the blood meal, bodies from freshly fed mosquitoes, and mosquito bodies collected 5 days post-infection was determined using quantitative reverse transcriptase polymerase chain reaction (qRT-PCR) and WNV specific primers. RT reactions were performed using Enhanced Avian Reverse Transcriptase (Sigma, St. Louis, MO) and quantitative real time PCR performed using SsoAdvanced SYBR Green Supermix (BIO-RAD, Hercules, CA) and following the included protocols. The standard curve was generated based on 10-fold serial dilutions of WNV and quantified by qRT-PCR as described above. WNV titer was determined from triplicate cycle threshold (Ct) values using Bio-Rad CFX manager software. The data was normalized by Log10 transformation and regression analysis was used to determine a qPCR-derived titer (Qpfu/ml).

### Differentially expressed gene analysis

The Sequencing Core of the University of Miami Miller School of Medicine provided the raw data from the Illumina analysis. The Illumina raw data was subjected to RNAseq analysis by the Center for Genetic Epidemiology and Statistical Genetics Core that is part of the Hussman Institute for Human Genomics at the University of Miami to determine differentially expressed genes between two populations after WNV oral infection (Table 1). Samples were barcoded to allow for multiplexing of 3 samples per HiSeq2000 PE100 lane. The sequencing data is deposited in Short Read Archive at NCBI (Accession number SRS515667, SRS516159). The mapped reads were assembled into transcripts and the abundance and differential expression of the reads between the two populations, CQV and CQG, measured by Cufflinks and Cuffdiff [33, 34].

The differentially expressed genes from RNAseq data analysis were validated by Real-Time quantitative RT-PCR. A total of 8 genes were randomly selected from the differentially expressed transcript list from the Cuffdiff analysis (q < 0.05, Table 3). The primer sets were designed (Integrated DNA Technologies), total RNA was extracted by Trizol from female mosquito bodies, which were prepared in the same way as described above and three biological replications were used for the validation. cDNA synthesis was carried out using the Enhanced Avian HS RT-PCR kit (Sigma, Saint Louis, MO) with reverse transcription completed using oligo dT. Actin protein gene was used as a control endogenous gene and the randomly selected genes were quantified with Real-Time quantitative RT-PCR.

The functions of the putative proteins encoded by the *Cx. quinquefasciatus* transcripts are theoretical and based on nucleotide sequence similarity to other orthologous organisms as described in the literature. Taking this into account, Gene Ontology analysis was performed (BioMart.org, Vector Base.org).

### Ovary development study

We used the same aged females from each population as previously described. After bloodfeeding with/without WNV, ovaries were dissected (10, 12, 14, 16, 18, 24, 36, and 48 hours post blood-feeding) and fixed on glass slides with one drop of Canadian balsam and cover glass added. At least 10 females were used for ovary dissections in each time point per population with or without WNV infection. The fixed samples were visualized under an Olympus SZX12 microscope and pictures taken under the microscope with 16 times magnification. The pictures were analyzed with imageJ (from http://rsbweb.nih.gov/ij/) to determine the area occupied by each ovary. We conducted statistical analyses for ovary growth based on measurements of the size by visual inspection of the digital photograph (Figure 3). Analysis of variance (PROC GLM, SAS 9.22) was used to evaluate differences in ovary growth in each group. If significant differences were observed, then a Duncan test was used to determine differences in the means.
